# Biased Symptom Reporting and Antisocial Behaviour in Forensic Samples: A Weak Link

**DOI:** 10.1080/13218719.2016.1256017

**Published:** 2016-12-12

**Authors:** Alfons van Impelen, Harald Merckelbach, Isabella J. M. Niesten, Marko Jelicic, Benno Huhnt, Joost á Campo

**Affiliations:** a Department of Clinical Psychological Science, Maastricht University, The Netherlands; b Berlin, Germany; c Radix Forensic Psychiatric Hospital, Heerlen, The Netherlands

**Keywords:** antisocial personality disorder, malingering, psychopathy, response bias, symptom validity

## Abstract

In two studies (one with 57 forensic inpatients and one with 45 prisoners) the connection between biased symptom reporting and antisocial behaviour is explored. The findings are as follows: 1) the association between symptom over-reporting and antisocial features is a) present in self-report measures, but not in behavioural measures, and b) stronger in the punitive setting than in the therapeutic setting; and 2) participants who over-report symptoms a) are prone to attribute blame for their offence to mental disorders, and b) tend to report heightened levels of antisocial features, but the reverse is not true. The data provide little support for the inclusion of antisocial behaviour (i.e. antisocial personality disorder) as a signal of symptom over-reporting (i.e. malingering) in the *Diagnostic and Statistical Manual of Mental Disorders – Fifth Edition* (*DSM-5*). The empirical literature on symptom over-reporting and antisocial/psychopathic behaviour is discussed and it is argued that the utility of antisocial behaviour as an indicator of biased symptom reporting is unacceptably low.

## Introduction

Antisocial behaviour and deceptive tendencies are considered to be core characteristics of both antisocial personality disorder (ASPD; American Psychiatric Association, 1980, 2000, 2013) and psychopathy (Cleckley, 1941, 1988; Hare, 1991, 2003; Hare, Forth, & Hart, 1989). A specific variant of deceptive behaviour is referred to in the *Diagnostic and Statistical Manual of Mental Disorders* *–* *Fifth Edition* (*DSM-5*; American Psychiatric Association, 2013) as *malingering*: ‘The intentional production of false or grossly exaggerated physical or psychological symptoms, motivated by external incentives’ (American Psychiatric Association, 2013, p. 726).

Precisely because malingering is a form of deception – and because such behaviour infringes social norms – the idea that malingering is strongly associated with ASPD and psychopathy has great intuitive appeal. Accordingly, the *DSM* – from its third edition onwards – assumes that antisocial behaviour is intimately linked to malingering. Indeed, the *DSM-5* lists the presence of ASPD among the indications that warrant heightened suspicion of malingering. However, in contrast to its *prima facie* plausibility, the empirical support for this idea is weak. In fact, early reviews (Clark, 1997; DeMatteo & Edens, 2006) concluded that there is a paucity of studies demonstrating the link between antisocial behaviour and malingering. More recently, Niesten, Nentjes, Merckelbach, and Bernstein (2015) conducted a systematic search by means of several databases and confirmed the mixed findings in this domain: of the seven studies found that explore whether psychopathic and antisocial behaviour are related to symptom over-reporting, four found an association – albeit a relatively weak one – (e.g. Heinze & Vess, 2005; Kucharski, Duncan, Egan, & Falkenbach, 2006), one did not find a relation (Pierson, Rosenfeld, Green, & Belfi, 2011), and two produced conflicting results (Cima & van Oorsouw, 2013; Sumanti, Boone, Savodnik, & Gorsuch, 2006). Furthermore, a recent meta-analysis of the relation between distorted response styles and self-reported psychopathic traits revealed a medium association between symptom over-reporting (i.e. malingering) and the antisocial lifestyle factor (95% CI of weighted mean effect size: [.23, .40]), but not the manipulative, callous personality factor (95% CI: [.00, .14]) of psychopathy (Ray et al., 2013).

Some authors (e.g. MacNeil & Holden, 2006) have speculated that high levels of psychopathy are associated with greater *proficiency* in successful (i.e. undetected) faking. Even if psychopathic traits would confer no aptitude for malingering directly, they could still lead to gains in proficiency through practice, as they may prompt individuals to engage in malingering more frequently. However, there is hardly any support for the hypothesis that antisocial and psychopathic traits foster the *ability* to malinger (e.g. Marion et al., 2013; for an overview, see Niesten et al., 2015).

Distorted symptom reports during clinical assessment are not limited to the exaggeration of symptoms; they may also take the form of the denial of such symptoms, as well as the exaggeration of positive qualities or indicators of good health (i.e. social desirability). Much like malingering, symptom under-reporting and social desirability are principally deceptive and manipulative in nature. Hence, it stands to reason that if antisocial and psychopathic traits predispose to symptom over-reporting (i.e. malingering), these features may also predispose to symptom under-reporting.

The extant literature about the relation between symptom under-reporting and social desirability, on the one hand, and antisocial and psychopathic traits, on the other, is even scarcer than that on symptom over-reporting and antisocial or psychopathic traits. The investigation of Niesten et al. (2015) only yielded two studies that are directly relevant: Cima, van Bergen, and Kremer (2008), who found no association, and Freeman and Samson (2012), who found psychopathy to be associated with *less* symptom under-reporting. Additionally, the meta-analysis of Ray et al. (2013) showed that the antisocial lifestyle factor (95% CI of weighted mean effect size: [−.25, −.06]), but not the manipulative, callous personality factor (95% CI: [−.06, .05]) of psychopathy is negatively related to symptom under-reporting and social desirability.

One explanation for the conflicting findings in this research domain is that the links between biased symptom reporting and antisocial features are context dependent. Thus, prison inmates may feign psychiatric symptoms in an attempt to be transferred from prison to the relatively mild conditions of a forensic psychiatric hospital. Once in a psychiatric hospital, they may exaggerate their mental fitness to reduce mandatory treatment. Similarly, defendants may feign symptoms in an attempt to reduce their criminal responsibility, yet employ symptom under-reporting and social desirability post-conviction to obtain privileges, probation, or parole. Likewise, plaintiffs may feign particular symptom constellations (e.g. post-traumatic stress, burnout, chronic pains) in the service of a compensation claim, while simultaneously denying genuine problems (e.g. substance use, impulsivity, compulsivity) to make a favourable impression on judicial decision-makers (e.g. Cima & van Oorsouw 2013; Niesten et al., 2015).

The idea – as endorsed by the *DSM-5* – that antisocial behaviour is associated with symptom over-reporting is further examined in two studies. Unlike other studies in this field, symptom under-reporting (Study 1) and social desirability (Study 2) are also examined, because such behaviour is no less deceptive than symptom over-reporting, and thus – at face value – it is equally plausible for it to be related to antisocial and psychopathic behaviour. To explore the idea that the relations between biased symptom reporting and antisocial features are context dependent, a therapeutic forensic setting (Study 1) is contrasted with a punitive forensic setting (Study 2).

## Study 1

The aim of Study 1 is to investigate the relationship between biased symptom reporting (i.e. over-reporting and under-reporting) and antisocial behaviour (measured by institutional misbehaviour and ASPD diagnoses) in a forensic psychiatric context. Given the mixed findings in the literature mentioned earlier, it was expected that little to no relationship between antisocial behaviour and biased symptom reporting would be found. The therapeutic environment of the psychiatric hospital (in which treatment progress leads to privileges such as furloughs and access to accommodation for recreational activities) created the anticipation of a higher rate of symptom under-reporting than symptom over-reporting. Prior to data collection, ethical approval was obtained from the Ethical Committee of the Faculty of Psychology and Neuroscience, Maastricht University, and from Radix Forensic Psychiatric Hospital, Heerlen, The Netherlands.

## Method

### Participants

Participants were recruited from Radix, a medium security forensic psychiatric hospital in the Netherlands that admits patients post-trial. Confinement there is in lieu of regular punishment, but not mandatory; patients can opt for incarceration in a penitentiary (and also opt for this during their stay). All patients undergo psychological and neuropsychological assessment upon admission to establish diagnoses as laid out in the *DSM-5*. In addition to individually tailored treatment for their psychopathology, patients are obliged to partake in group therapies aimed at resocialisation and recidivism risk reduction. Patients remain in treatment until they are ready to re-enter society or until their prison term ends.

Treatment supervisors provided the names of patients they deemed fit to participate and these patients were then invited to take part in the study. Exclusion criteria (as determined by treatment supervisors) included insufficient command of the Dutch language, extreme symptoms of drug withdrawal, severe mental instability due to psychosis, or deficient mental abilities owing to severe intellectual disability. On these grounds, 25 patients were not approached. The majority of these patients were found to be unfit to participate because of substance withdrawal symptoms, severe intellectual disability, or psychosis. Furthermore, as participation was voluntary and did not yield any rewards, 13 eligible patients chose not to participate. Another 3 patients absconded before behavioural observations were completed, and 1 patient chose to terminate his participation shortly after starting the first test.

In total, 57 male inpatients aged 19 to 54 years (*M* = 40.0, *SD* = 9.1) completed the study. The mean IQ was 88.2 (*SD* = 14.6, range = 61–140; IQ scores are missing for 2 participants). IQ data were gathered from patient records, which contained Wechsler Adult Intelligence Scale – Fourth Edition (WAIS-IV; Wechsler, 2008) protocols. The majority of participants are Caucasian (79%, *n* = 45) and all except one (98%, *n* = 56) had been diagnosed with one or multiple substance disorders. Furthermore, 28% (*n* = 16) had received a diagnosis of other specified personality disorder, 16% (*n* = 9) had been diagnosed with ASPD, 14% (*n* = 8) had been diagnosed with autism spectrum disorder, 7% (*n* = 4) had been diagnosed with attention-deficit hyperactivity disorder (ADHD), 7% (*n* = 4) had been diagnosed with schizophrenia, and 19% (*n* = 11) received no diagnosis other than substance disorder.

### Measures

#### 

##### The Structured Inventory of Malingered Symptomatology (SIMS)

The SIMS (Smith & Burger, 1997; see Merckelbach & Smith, 2003 for the Dutch translation) is a symptom validity test that assesses a broad spectrum of feigned and exaggerated symptoms. The SIMS consists of 75 true–false items, which constitute five subscales that target feigned depression, psychosis, neurologic impairment, memory dysfunction, and low intelligence. The items mostly refer to bizarre experiences and atypical symptoms such as ‘I have difficulty recognising written and spoken words’ and ‘When I can't remember something, hints do not help’. The number of endorsed symptoms is summed so as to obtain a total SIMS score. The SIMS does not require a high reading level (i.e. Flesch–Kincaid Scale 5.3 suffices; Smith, 2008). The internal consistency of the SIMS is reasonable (Cronbach's alpha coefficients of .72 found by Merckelbach & Smith, 2003, and .92 to .94 by Rogers, Robinson, & Gillard, 2014). Both studies with experimental simulators and studies with identified malingerers (i.e. known-groups studies) have yielded acceptable diagnostic accuracy parameters, with sensitivity circling around .91 and specificity around .65 for a cut-off of >16 (for a detailed overview, see van Impelen, Merckelbach, Jelicic, & Merten, 2014).

##### The Supernormality Scale (SS)

The SS (Cima et al., 2003) is a self-report instrument that has been developed as a research tool for assessing symptom under-reporting. It consists of 37 true–false items, of which 21 items comprise a supernormality subscale (measuring minimisation of mild psychopathological phenomena), 11 items comprise a social desirability subscale, and 5 items are bogus. An illustrative supernormality item is ‘I have my problems under full control’, and an example of a social desirability item is ‘I try to help everybody who has problems’. Endorsement of supernormality and social desirability items is summed so as to obtain a total SS score. While the internal consistency (Cronbach's alpha = .86) and test–retest reliability (*r* = .90) of the SS are satisfactory, the diagnostic accuracy indices are meagre, with the sensitivity and specificity being .74 and .42 for a >14 cut-off score, .58 and .67 for a >17 cut-off score, and .28 and .93 for a >21 cut-off score, respectively (Cima et al., 2003). However, the SS does possess moderate predictive validity, as undergraduate students instructed to imagine that they were offenders who opted for parole and who therefore engaged in faking good exhibited statistically higher scores compared to control individuals (Cima et al., 2003).

##### The Levenson Self-Report Psychopathy Scale (LSRPS)

The LSRPS (Levenson, Kiehl, & Fitzpatrick, 1995) is a 26-item self-report instrument that assesses traits associated with a callous and manipulative orientation towards others (i.e. primary psychopathy) and with a disinhibited and antisocial lifestyle (i.e. secondary psychopathy). Items are scored on four-point Likert scales (where 1 = *strongly disagree* and 4 = *strongly agree*). A total score that is reflective of psychopathic traits can be calculated by recoding some items and then summing all scores. Total scores of >57 are considered to be ‘high’ (Brinkley, Schmitt, Smith, & Newman, 2001). A representative item from the primary psychopathy subscale is ‘[i]n today's world, I feel justified in doing anything I can get away with to succeed’, whereas an illustrative item from the secondary psychopathy scale is ‘I have been in a lot of shouting matches with other people’. While the LSRPS was originally designed to assess psychopathic traits in non-institutionalised samples, it has been employed successfully in large forensic samples (Brinkley et al., 2001; Walters, Brinkley, Magaletta, & Diamond, 2008). The internal consistency of the total and primary psychopathy scale is adequate (Cronbach's alpha = .82–.84), whereas that of the secondary psychopathy scale is moderate (Cronbach's alpha = .63–.68; Levenson et al., 1995; Lynam, Whiteside, & Jones, 1999).

##### The Social Dysfunction and Aggression Scale-11 (SDAS-11)

The SDAS-11 (Wistedt et al., 1990) is an 11-item behavioural observation scale that was developed to measure social dysfunction and aggression in psychiatric inpatients. The SDAS-11 is scored over a longer time interval, with one-week intervals between successive ratings. It consists of 9 items covering outward aggression and social dysfunction (e.g. irritability, negativism, verbal and physical aggression) and 2 items covering inward aggression (i.e. self-harm), with each item including a five-point scoring scale (ranging from 0 = *not present* to 4 = *severe*). The outward and inward items are not inter-correlated and the internal consistency is acceptable (Cronbach's alpha = .79; Wistedt et al., 1990). In the current study, SDAS-11 items were used to evaluate the daily nurse observation records of each participant. More specifically, for each participant, all records of a six-week period were selected and scored in terms of indications for the presence of SDAS-11 items. To explore the reliability of this procedure, a random set of ten one-week records from 10 patients were selected and evaluated by the first author and another rater who was also blind to the symptom validity status associated with each record. The Spearman rank order correlation between the two raters is .79.

### Procedure

Seated in a small therapy room on their own ward, participants first gave written informed consent that was also verbally communicated to them. Next, participants completed the test battery, which included – in counterbalanced order – the SIMS, the SS, and for a subsample (*n* = 25) also the LSRPS. The test battery also included an instrument that is not addressed in the current study: the Vocabulary and Abstraction subtests of the Malingering Scale (Schretlen & Arkowitz, 1990), which are cognitive paper-and-pencil tasks that measure underperformance. After participants had completed the test battery, their engagement in institutional misbehaviour was monitored for a period of six weeks. The monitoring of participants was achieved through the close examination of patient records, which were maintained on a daily basis by nursing staff, therapists, psychologists, physicians, and treatment supervisors. The patient records that were used for the present study contain reports of daily activities and detailed accounts of clinically relevant activities and behaviour, such as social functioning, treatment progress, and physical and emotional well-being. As such, the patient records contain ample information for completing the SDAS-11 items. For each participant, the scores on the test battery were calculated only after scores on the SDAS-11 had been obtained, thus reducing experimenter bias during the evaluation of patient records with SDAS-11 items.

## Results

Table 1 summarises the mean scores on the psychometric instruments and also gives the proportion of patients who scored above cut-off points, as well as the prevalence of the most frequent diagnoses among these patients. As can be seen, symptom under-reporting (as indexed by the SS) is more than twice as prevalent as symptom over-reporting (as indexed by the SIMS).

**Table 1.** Summary of means, *SD*s, 95% confidence intervals and prevalence rates of diagnoses and cut-off failures in the forensic patient sample (*n* = 57).

	M (*SD*)	95% CI	Percentage exceeding cut-off	Percentage of most frequent diagnoses of patients scoring > cut-off
SIMS	8.6 (6.6)	[6.8, 10.3]	9% (5 out of 57) >16	60% IQ <75, 40% OSPD
SS	16.4 (6.5)	[14.7, 18.2]	23% (13 out of 57) >21	38% OSPD, 23% IQ <75
LSRPS	52.6 (9.6)	[48.6, 56.6]	28% (7 out of 25) >57	No diagnoses with a count > 1
SDAS-11	17.1 (12.5)	[13.8, 20.4]	16% (8 out of 57) >1.5 *SD*s	38% IQ <75, 25% OSPD

Note: LSRPS = Levenson Self-Report Psychopathy scale; OSPD = other specified personality disorder; SDAS-11 = Social Dysfunction and Aggression Scale-11; SIMS = Structured Inventory of Malingered Symptomatology; SS = Supernormality Scale.

The most frequent diagnoses among participants who failed the SIMS cut-off score (*n* = 5, 9%) are an IQ of <75 (60%) and other specified personality disorders (OSPDs; 40%). Among participants who failed the SS cut-off score (*n* = 13, 23%), the most frequent diagnoses are OSPDs (38%) and an IQ of <75 (23%). Furthermore, two participants produced a significantly outlying score (>2 *SD*s) on the LSRPS; they were diagnosed with ASPD and autism spectrum disorder, respectively. The group of participants who scored beyond 1.5 *SD*s on the SDAS-11 (*n* = 8, 14%) is diverse with regard to diagnoses: three participants (38%) had an IQ of <75 and two (25%) had an OSPD diagnosis. There is no overlap between the group of participants who exceeded the cut-off score of the SIMS and the group who exceeded the cut-off scores of the SS or the LSRPS. Of the thirteen participants who scored above the cut-off on the SS, three (23%) have SDAS-11 scores of 1.5 *SD*s above the mean and two have an LSRPS score that surpasses the cut-off of >57. Interestingly, the mean SDAS-11 score of ASPD patients does not differ statistically from that of the other patients (15.4 vs 17.3), *t*(55) = 0.3, *p* = .75, a result that squares with the recent finding of Edens, Kelley, Lilienfeld, Skeem, and Douglas (2015) that ASPD has no predictive value for institutional misconduct.

To examine the relation between antisocial features and biased symptom reporting, binary contingency tables were computed for all antisocial behaviour indices (i.e. psychopathic traits; LSRPS scores >57, institutional misbehaviour; SDAS-11 scores > 1.5 *SD*s above the mean, and ASPD diagnoses) and biased symptom reports (i.e. symptom over-reporting; SIMS scores >16, and symptom under-reporting; SS scores >21). Fisher's exact tests indicate that biased symptom reporting is not associated with psychopathic traits, institutional misbehaviour, or ASPD diagnoses (all *p*s > .05).

As another approach to data analysis, Pearson product–moment correlations among the various measures were calculated (Table 2). Neither self-reported psychopathic traits (LSRPS) nor institutional misbehaviour (SDAS-11) were found to be strongly related to symptom over-reporting (SIMS) or under-reporting (SS). Institutional misbehaviour (SDAS-11) was found to be unrelated to symptom over-reporting (SIMS) or under-reporting (SS), whereas self-reported psychopathic traits (LSRPS) seem to be moderately associated with symptom over-reporting (SIMS), but not under-reporting (SS). Age is not related to any of the measures; IQ is related only to symptom under-reporting, with those with higher IQs predisposed towards less under-reporting, *r* = −.30 [−.52, −.04], *p* = .03, two-tailed.

**Table 2.** Pearson product–moment correlations and 95% confidence intervals for the Study 1 data.

**Measure**	**1 SIMS**	**2 SS**	**3 LSRPS**
**2 SS**	−.28* [−.50, −.02]	–	
**3 LSRPS**ᵃ	.31 [−.09, .63]	−.10 [−.30, .48]	–
**4 SDAS-11**	−.04 [−.30, .22]	.00 [−.26, .26]	.27 [−14, .60]

Note: *p < .05, two-tailed; ᵃ*n* = 25. LSRPS = Levenson Self-Report Psychopathy Scale; SDAS-11 = Social Dysfunction and Aggression Scale-11; SIMS = Structured Inventory of Malingered Symptomatology; SS = Supernormality Scale.

Consistent with the correlational analyses, multiple linear regression analyses indicate that neither self-reported psychopathic traits (LSRPS) nor institutional misbehaviour (SDAS-11) are predictive of symptom over-reporting (SIMS), *F*(2, 22) = 1.70, *p* = .21, or symptom under-reporting (SS), *F*(2, 22) = 0.40, *p* = .67. Analyses including the scores on the subscales of the SIMS, SS, and LSRPS did not yield additional information. In sum, no association was found between institutional misbehaviour and either form of biased symptom reporting, nor is there a relation between biased symptom reporting and ASPD or self-reported psychopathy (yet the latter is based on *n* = 25).

Next, groups were formed based on the temporal trends of the SDAS-11 scores; one group had scores that increased over time (*n* = 23), one group had scores that remained relatively stable (*n* = 26), and one group had scores that decreased over time (*n* = 8). The three groups were then compared with regard to biased symptom-reporting (i.e. frequency of individuals scoring above the SIMS or SS cut-offs). Table 3 shows the patterns. Fisher's exact tests yielded no significant results (all *p*s > .05), which implies that institutional misbehaviour is in no way related to symptom over-reporting or under-reporting.

**Table 3.** Numbers of forensic patients (*n* = 57) with increasing, constant, or decreasing antisocial behaviour who over-report or under-report symptoms.

	**Over-reporting (SIMS)**		**Under-reporting (SS)**	
**SDAS-11**	**Below cut-off (≤16)**	**Above cut-off (>16)**	**Below cut-off (≤21)**	**Above cut-off (>21)**
**Increasing**	21 (37%)	2 (4%)	18 (32%)	5 (9%)
**Stable**	24 (42%)	2 (4%)	20 (35%)	6 (11%)
**Decreasing**	7 (12%)	1 (2%)	6 (11%)	2 (4%)

Note: SDAS-11 = Social Dysfunction and Aggression Scale-11; SIMS = Structured Inventory of Malingered Symptomatology; SS = Supernormality Scale.

## Discussion

The prevalence of symptom over-reporting in this sample of forensic psychiatric inpatients is relatively low (9%) compared with estimates that can be found in the literature (cf. 19%: Mittenberg, Patton, Canyock, & Condit, 2002; 32%: Pollock, Quigley, Norley, & Bashford, 1997). The participants in the present study were recruited from a forensic psychiatric hospital where patients are admitted once their sentences have been passed, and in which patients have relatively few apparent external incentives to over-report symptoms – in fact, doing so may even result in delayed furloughs and prolonged stays. This might explain why symptom under-reporting was more than twice as prevalent as symptom over-reporting in the present sample (23% vs 9%). Additionally, the prevalence of symptom over-reporting may have been low because of selection bias: treatment supervisors prohibited the inclusion of patients they deemed too disordered to participate. It may be that a portion of these patients exaggerated their pathology (and would have over-reported symptoms had they participated).

The prevalence of ASPD (16%) and antisocial behaviour is low as well. A large portion of patients’ SDAS-11 scores are explained by irritability, negativism, mild resentment, and moderate verbal aggression. Thus, participants engaged almost exclusively in mild disruptive behaviour; none engaged in serious physical violence, self-harm, or severe verbal aggression. The relative absence of gravely disruptive behaviour is likely due to several factors. First, it may have to do with the focus on treatment and the consequently comprehensive and constant implementation of a zero tolerance policy on all wards, which have a staff to patient ratio of at least 1:6 (usually 1:4). Second, it may be related to the considerable weight of the consequences of misbehaviour, which typically include delayed or revoked furloughs, prolonged stays, or – in severe cases – relocation to a penal institution.

## Study 2

This study examines the relation between biased symptom reporting and antisocial behaviour in a punitive forensic setting. Additionally, several types of blame attribution and excuse-making are assessed. Niesten et al. (2015) report an interesting difference between forensic psychiatric patients and prisoners with respect to symptom over-reporting and under-reporting. More specifically, they found that both types of distorted symptom reporting are higher in the latter group, presumably because the incentives to distort symptoms are higher in that context. With this in mind, more symptom over-reporting was expected in the punitive setting of Study 2 than in the therapeutic setting of Study 1, yet it was also predicted that the relationship between biased symptom reporting and antisocial behaviour would be similarly small. Cima et al. (2003) observe that symptom under-reporting in a forensic sample is related to the tendency to blame external conditions or others for their crimes. To extend this work, it was decided to test whether symptom over-reporting is associated with excuse-making and blame attribution to mental disorders. Approval was obtained from the standing Ethical Committee of the faculty of Psychology and Neuroscience, Maastricht University, and the Youth Prison of Berlin (*Jugendstrafanstalt Berlin*), Germany.

## Method

### Participants

Inmates of an all-male youth prison in Berlin were proffered a brief description of the study and invited to participate in two test sessions without compensation. Insufficient literacy and command of the German language are the only exclusion criteria. A total of 65 inmates agreed to participate and completed the first session, but only 45 of those completed the second session, which took place two to three weeks later. Reasons for dropping out included completion of the prison sentence, relocation to another facility, and lack of interest in the second session. The majority of the final sample (*n* = 45) were sentenced prisoners, and 4 (9%) were on remand. The mean age was 20.7 years (*SD* = 1.7, range = 18–24).

### Measures

Antisocial and delinquent behaviour was measured with several proxies: length of prison sentences (in years), number of incurred disciplinary actions (coded as a continuous variable), and classification as ‘intensive offender’ (yes/no). Intensive offender (*Intensivtäter*) is a term used in Germany to designate juveniles whose delinquency is serious and repetitive. Although there are no formal definitions or criteria to establish intensive offending, the term is commonplace in the German justice system. The SIMS and the LSRPS were used (see Study 1 for details), along with two other instruments. The test battery also included a measure of symptom overreporting that was not used in the analyses below (the recently developed *Self-Report Symptom Inventory*; SRSI; Merten, Merckelbach, Giger, & Stevens, 2016).

#### 

##### The Social Desirability Scale-5 (SDS-5)

The SDS-5 consists of five modified items from the Social Desirability Scale-17 (SDS-17; Stöber, 2001). The internal consistency (Cronbach's alpha = .80) and test–retest reliability (*r* = .82) of the SDS-17 are adequate. The SDS-5 was embedded in the LSRPS. Therefore, the original true/false format is replaced with a four-point scale (where 1 = *I do not at all agree* and 4 = *I fully agree*).

##### The Revised Gudjonsson Blame Attribution Inventory (BAI)

The BAI (Gudjonsson & Singh, 1989; for the German translation, see Cima et al., 2006) contains 42 items that tap into three independent dimensions of blame attribution for criminal offences: external attribution (i.e. blaming transgressions on social environments, victims, or society; Cronbach's alpha = .77), mental-element attribution (i.e. placing blame on mental disorders or insufficient self-control; Cronbach's alpha = .79), and guilt-feeling attribution (i.e. feeling remorse or regret about offences; Cronbach's alpha = .81). Items consist of first-person statements that are evaluated on a five-point scale (where 0 = *I do not at all agree* and 4 = *I fully agree*). Representative items include ‘I did not deserve to be caught for the crime I committed’ (external attribution), ‘I would certainly not have committed the crime I did if I had been mentally well’ (mental-element attribution), and ‘I have no serious regrets about what I did’ (guilt-feeling attribution).

### Procedure

Two sessions were undertaken, the first containing the informed consent form followed by administration of the SRSI. The second session (which took place two to three weeks later) consisted of administration of the SIMS, the BAI, and the LSRPS with the SDS-5 items. Participants were told that the instruments measured personality characteristics and psychological problems.

## Results

Table 4 presents means scores of the sample on the various measures, as well as the proportion of prisoners whose scores exceed the associated cut-offs. As can be seen, 13% (*n* = 6) of the participants failed the SIMS, which is only slightly higher than the failure rate in Study 1 (9%). More than half of the sample scored above the cut-off on the LSRPS (56%), which is considerably higher than the proportion with extreme LSRPS scores in Study 1 (28%).

**Table 4.** Summary of means, *SD*s, 95% confidence intervals and prevalence rates of cut-off failures in the forensic punitive sample (*n* = 45).

	M (*SD*)	95% CI	Percentage exceeding cut-off
SIMS	10.6 (5.2)	[9.1, 12.2]	13% (6 out of 45) >16
SDS-5	16.7 (2.7)	[15.9, 17.5]	N/A
LSRPS	58.9 (9.3)	[56.1, 61.7]	56% (25 out of 45) >57
BAI External	29.5 (8.5)	[26.9, 32.0]	N/A
BAI Mental	25.0 (6.6)	[23.0, 27.0]	N/A
BAI Guilt	54.6 (11.7)	[51.1, 58.2]	N/A
Prison term (years)	2.5 (1.4)	[2.1, 2.9]	N/A
Punitive actions	0.5 (0.9)	[0.3, 0.8]	N/A
Intensive offender	N/A	N/A	22% (10 out of 45)

Note: BAI = Revised Gudjonsson Blame Attribution Inventory; LSRPS = Levenson Self-Report Psychopathy Scale; Punitive actions = relative number of punitive actions taken against participants; SDS-5 = Social Desirability Scale-5; SIMS = Structured Inventory of Malingered Symptomatology.

Table 5 displays the correlations among the various measures. Symptom over-reporting (SIMS) correlates positively with self-reported psychopathic traits as measured by the LSRPS and negatively with social desirability as indexed by the SDS-5. Symptom over-reporting is also related to blame attribution to external factors such as social environments, victims, or society (BAI External) and to mental disorders (BAI Mental). However, symptom over-reporting is not statistically related to behavioural proxies of antisocial behaviour (i.e. sentence length, number of incurred disciplinary actions, or classification as intensive offender). Also, the four participants on remand did not produce SIMS or LSRPS scores above the respective cut-offs, despite two of them being considered intensive offenders. Analyses containing subscale scores of the SIMS and LSRPS revealed that primary psychopathy scores are moderately related to age; older participants reported more callous and manipulative demeanour, *r* = .38 [.10, .61], *p* < .01, two-tailed.

**Table 5:** Pearson product–moment correlations and 95% confidence intervals for the Study 2 data.

		1 SIMS	2 SDS-5	3 LSRPS	4 BAI External	5 BAI Mental	6 BAI Guilt	7 Prison term (years)	8 Punitive actions
2	SDS-5	−.42** [−.63, −.14]	–						
3	LSRPS	.60** [.37, .76]	−.50** [−.69, −.24]	–					
4	BAI External	.35* [.06, .59]	−.27 [−.53, .03]	.41** [.13, .63]	–				
5	BAI Mental	.43** [.16, .65]	−.12 [−.40, .18]	.24 [−.06, .50]	.18 [−.12, .46]	–			
6	BAI Guilt	−.09 [−.38, .21]	.21 [−.10, .47]	−.26 [−.52, .04]	−.50** [−.70, −.24]	.33* [.04, .57]	–		
7	Prison term (years)	.23 [−.08, .49]	−.17 [−.45, .13]	.09 [−.21, .38]	−.11 [−.39, .19]	.22 [−.09, .48]	−.01 [−.31, .29]	–	
8	Punitive actions	−.05 [−.34, .25]	.16 [−.15, .43]	.01 [−.28, .30]	−.05 [−.34, .25]	−.01 [−.30, .28]	.08 [−.22, .36]	.13 [−.17, .41]	–
9	Intensive offender	−.14 [−.42, .16]	.02 [−.28, .31]	.02 [−.28, .31]	−.02 [−.31, .28]	−.03 [−.32, .27]	.04 [−.26, .33]	.33* [−.04, .57]	−.02 [−.31, .28]

Note: *p < .05, two-tailed; **p < .01, two-tailed. BAI = Revised Gudjonsson Blame Attribution Inventory; LSRPS = Levenson Self-Report Psychopathy Scale; Punitive actions = relative number of punitive actions taken against participants; SDS-5 = Social Desirability Scale-5; SIMS = Structured Inventory of Malingered Symptomatology.

Multiple linear regression analyses revealed that self-reported psychopathic traits (LSRPS; *B* = .30, *p* < .01), and to a lesser extent blame attribution to mental disorders (BAI Mental; *B* = .24, *p* = .01), are predictive of symptom over-reporting, *R*^2^ = .46, *F*(2, 41) = 17.69, *p* < .01. Self-reported psychopathic traits (LSRPS; *B* = −.14, *p* < .01) are moderately predictive of a less socially desirable response style (SDS-5), *R*^2^ = .24, *F*(1, 42) = 13.58, *p* < .01.

## Discussion

Analogous to Study 1, the second study relies on a self-report instrument to assess psychopathy. Self-report measures, however, may not be suitable for quantifying psychopathic traits because such traits include the inclination to deceive and manipulate (for a meta-analytic review, see Ray et al., 2013; for a qualitative review, see Kelsey, Rogers, & Robinson, 2014). This concern is addressed through the inclusion of a concise measure of socially desirable responding (i.e. the SDS-5). A substantial negative correlation was found between self-reported psychopathy and socially desirable response bias: participants who scored higher on the LSRPS generally scored lower on the SDS-5. This result is reminiscent of Niesten et al. (2015), who found a negative correlation between faking good and psychopathy in their forensic sample. Arguably, the most salient interpretation is that participants with more psychopathic traits and/or more severe psychopathic traits hold (and report) attitudes that are less socially desirable.

An alternative explanation is that many participants who answered in a highly socially desirable manner under-reported psychopathic attitudes and behaviours. A Pearson's chi-square analysis of the SDS-5 (>18) and LSRPS (>57) groups revealed that 50% of participants who produced LSRPS scores below the cut-off displayed a highly socially desirable response bias, whereas this is the case for only 12% of the participants who scored above the cut-off on the LSRPS. Put differently, the participants who answered in a highly socially desirable manner were 7.3 times more likely (compared with participants who had a less socially desirable response bias) to score low on psychopathic attitudes and behaviours. Thus, the relatively low psychopathy scores of some participants might be the result of an intentionally distorted response style (but see Watts et al., 2015). Unfortunately, this drawback extends to all measures that rely on self-report (e.g. the BAI), although measures that are less transparent (e.g. the SIMS) may be more robust.

A socially desirable response style also correlates negatively with symptom over-reporting (SIMS scores). It can be argued that this is counterintuitive by appealing to the common denominator of socially desirable response bias and symptom over-reporting; both behaviours amount to deception. On the other hand, psychopathology is not typically perceived as socially desirable (unless it constitutes grounds for excuse-making) and, as such, it may be expected that participants who are keen to make a good impression will not over-report psychopathological symptoms. This is exactly what the data shows; there is a subgroup of participants (*n* = 8) who seem particularly determined to make a good impression by answering in a socially desirable way. This group scored low on both the LSRPS and the BAI External scale (which both comprise socially undesirable items) and refrained from excessive endorsement of psychopathology as measured by the SIMS.

One could argue that the number of incurred disciplinary actions, classification as intensive offender, and particularly the length of prison sentences are questionable as proxies for antisocial behaviour – let alone for ASPD. While such criticism is well founded (Edens et al., 2015), it does not detract from the aura of antisocial attitudes that these proxies possess, or from the relevance of these proxies to clinical and legal decision-making about inmates and forensic patients. When forming professional opinions of individuals (i.e. opinions that have to be substantiated), decision-makers – from psychiatrists and clinical psychologists to judges and probation officers – rely on quantitative indicators, such as the number of disciplinary actions and classification as an intensive offender. Therefore, these indicators warrant inclusion when testing the idea that antisocial demeanour predisposes to biased symptom reporting.

## General Discussion

The findings of the present studies cast doubt on the intuitive assumption that antisocial behaviour is invariably related to biased symptom reporting. Moreover, they call into question two heuristic rules that – according to the *DSM*, since its third edition – give grounds for strong suspicion of malingering, namely, the presence of ASPD and a lack of compliance with treatment regimens. In Study 1, neither ASPD nor institutional misbehaviour (which included transgressions of treatment regimens) was found to be related to symptom over-reporting. In Study 2, symptom over-reporting was not linked to proxies of antisocial behaviour – notably length of prison sentence, number of institutional disciplinary actions, and classification as intensive offender.

In contrast to behavioural indices of antisocial features, in Study 2, self-report measures of such features were found to be related to symptom over-reporting. Analysis of the aggregated data of Studies 1 and 2 (*n* = 70) yields a correlation between symptom over-reporting (SIMS) and self-reported psychopathic traits (LSRPS) of *r* = .55 [.36, .70], *p* < .01, two-tailed. Endorsement of primary (i.e. callous and manipulative orientation toward others) as well as secondary (i.e. a disinhibited and antisocial lifestyle) psychopathic traits are related to heightened SIMS scores, *r* = .46 [.25, .63] and *r* = .49, [.29, .65], respectively, *p* < .01, two-tailed. This pattern seems to provide support for antisocial behaviour as an indicator of malingering. A comparison of actual test scores, however, reveals that the predictive value of self-reported psychopathic traits is limited: only 19% (6 out of 32) of those with elevated levels of psychopathic traits (LSRPS score >57) engaged in over-reporting of symptoms (SIMS score >16).

As previously stated, the association between symptom over-reporting and self-reported psychopathic traits is evident in Study 2, but not in Study 1. A possible reason for this discrepancy may be the limited number of LSRPS protocols that were obtained (*n* = 25) and the resulting lack of statistical power in Study 1, which is .34 (α = .05, *r* = .31, *n* = 25). This means that, when the correlation between symptom over-reporting and self-reported psychopathic traits is indeed moderate (i.e. .31), the probability of obtaining a significant result (i.e. *p* < .05) is only .34.

Another contributory factor to the discrepant results in the two studies may be the different frequencies of self-reported psychopathic traits. While the prevalence of symptom over-reporting is low in both studies (9% and 13%), the prevalence of self-reported psychopathic traits is substantially higher in Study 2 than in Study 1 (56% vs 28%). This may well be the result of contextual differences; the therapeutic setting of Study 1 is geared towards positive behavioural change and recidivism reduction, and actively suppresses the antisocial ‘survival of the toughest’ attitudes that typically exist within penal institutions such as the one investigated in Study 2 (Butler, 2008). Thus, a therapeutic climate may reduce antisocial features and/or deter disclosure of such features, which may curb the relation between self-reported psychopathic traits and symptom over-reporting.

In Study 2, the participants who over-reported symptoms attributed their offenses more to external factors such as social environments, victims, or society, and reported less remorse and regret for their wrongdoings relative to those who did not engage in symptom over-reporting. This is consistent with the positive relation between symptom over-reporting and self-reported psychopathic traits that also emerged in Study 2. Note, however, that these correlations all rely on self-report, and contradict the findings that are based on behavioural indices. For example, in Study 2, all participants whose SIMS score exceeded the cut-off (>16) produced a high LSRPS score (>57), yet none were deemed an intensive offender (point-biserial *r* SIMS intensive offender = −.14 [−.42, .16], *p* > .05, two-tailed). Moreover, these participants did not differ from the rest of the participants with regard to length of prison sentence and relative frequency of institutional disciplinary actions.

In Study 2, the attribution of criminal behaviour to mental disorders is associated with symptom over-reporting, *r* = .43, [.16, .65] *p* < .01, two-tailed, which lends some support to the hypothesis that symptom exaggeration can be a form of *post hoc* excuse-making for offences (Maruna & Mann, 2006). However, there is no difference in blame attribution to mental disorders between participants who scored beyond the SIMS cut-off (i.e. >16) and those who did not, *t*(42) = −1.41, *p* > .05. This suggests that symptom reporting generally coincides with attributions of blame to psychopathology, but that *excessive* symptom reporting does not necessarily coincide with *excessive* attributions of blame to psychopathology.

Given that the present studies only evaluated *if* participants engaged in symptom over-reporting and not *why* participants engaged in symptom over-reporting (i.e. the presence of external gains was not assessed), its findings are not a direct contradiction of those reported by, for example, Gacono, Meloy, Sheppard, Speth, and Roske (1995), who observed that offenders who malinger insanity exhibit more antisocial and psychopathic behaviour. It may be that relationships exist between specific forms of antisocial behaviour (e.g. aggressive narcissism and predatory violence) and specific forms of symptom over-reporting (e.g. successful malingering to obtain a ‘not guilty by reason of insanity’ verdict). However, the current findings suggest that the relationship between symptom over-reporting and antisocial behaviour is at best a specific one that does not hold for symptom over-reporting in general (i.e. as found in heterogeneous forensic populations).

It must be noted that the small sample sizes of the present studies prohibit the detection of subtle effects and small associations. While certainly interesting and worth investigating, such effects and associations are not relevant to the detection of symptom exaggeration and malingering, as is explained below. The modest sample sizes of the present studies suffice to detect effects that are sufficiently large to be diagnostically relevant (see the elaboration in note 1 below).

The considerable disparities among research findings should suffice to dissuade clinicians from using antisocial behaviour as a red flag for symptom over-reporting, as propagated by the *DSM-5*. Although some studies have found statistically significant correlations between symptom over-reporting and antisocial behaviour (Niesten et al., 2015), only one study yielded findings that are of a high predictive value (Gacono et al., 1995 found psychopathy to be a strong predictor of malingering insanity). The studies by Kucharski and colleagues are illustrative in this regard, observing a moderate effect of ASPD (Kucharski, Falkenbach, Egan, & Duncan, 2006) and psychopathy (Kucharski, Duncan, Egan, & Falkenbach, 2006) on symptom over-reporting (Cohen's *d =* .89 and .86, respectively, calculated using available information), but this does not produce satisfactory diagnostic values (accuracy = .52 and .43, respectively, calculated using available information). The point is that a positive relation between a predictor and a target condition does not constitute evidence for the utility of that predictor; it is the strength of the relation that determines the utility of the predictor.

The stronger the relation between a predictor and a target condition, the higher the predictive value of the predictor. The relational strength depends on two factors: joint occurrence and concurrent absence. That is, for a predictor to be predictively valuable, it must be present when the target condition is present (this corresponds to a *positive* predictive value) *and* absent when the target condition is absent (this corresponds to a *negative* predictive value). Considering the empirical literature, this is clearly not the case with symptom over-reporting and antisocial behaviour. For instance, in the study that found the strongest relation between symptom over-reporting and antisocial behaviour (Gacono et al., 1995), the prevalence of ASPD in the sample of *malingerers* was 100%, whereas the prevalence of ASPD in the sample of *non-malingerers* was 55%. Thus, all who engaged in malingering were diagnosed with ASPD, but not all who were diagnosed with ASPD engaged in malingering (only 64% did). The omnipresence of ASPD in the sample of malingerers may foster the intuitive allure of ASPD as a good predictor of malingering. Succumbing to this allure, however, amounts to committing the logical fallacy known as ‘affirming the consequent’. In the sample of Gacono et al. (1995), malingering is perfectly predictive of ASPD, yet the converse is not true; ASPD is only moderately predictive of malingering (64%).

A high prevalence of malingering among patients who are diagnosed with ASPD or psychopathy heightens vigilance with regard to malingering when patients have a record of antisocial behaviour, yet it may also decrease alertness when patients have no history of antisocial behaviour. While the former is warranted, the latter is tantamount to taking absence of evidence for evidence of absence (i.e. to committing the logical fallacy known as ‘denying the antecedent’). For example, in the forensic sample of Delain, Stafford, and Ben-Porath (2003), ASPD is fairly predictive of malingering (83%), but malingering is not predictive of ASPD (17%, calculated using available information).^1.^

In terms of diagnostic accuracy, affirming the consequent leads to false positive classifications (i.e. individuals who display antisocial behaviour but do not over-report symptoms are incorrectly considered to be malingerers). Similarly, denying the antecedent leads to false negative classifications (i.e. individuals who do not display antisocial behaviour but do engage in symptom over-reporting are incorrectly considered to be honest). The quantities of these errors depend on the prevalence of both symptom over-reporting among antisocial patients and antisocial behaviour among over-reporting patients. Few studies have assessed these prevalence rates, and even fewer still have looked into *both* prevalence rates among their samples. Table 6 displays prevalence rates of symptom over-reporting among patients with ASPD or psychopathy (and vice versa) and corresponding percentages of incorrect predictions when antisocial behaviour is used as predictor of symptom over-reporting. In addition to the two present studies, only studies that found a statistically significant relationship between symptom over-reporting and antisocial behaviour^2.^ are included in Table 6. The prevalence of symptom over-reporting among antisocial patients ranges from 11% to 90%, whereas the prevalence of antisocial behaviour among over-reporting patients varies between 17% and 100%.

**Table 6.** Prevalence rates and corresponding percentages of incorrect predictions of symptom over-reporting among antisocial patients and antisocial behaviour among over-reporting patients.

**Authors**	**Prevalence of symptomover-reportingamong antisocial patients**	**False positive predictions**	**Prevalence of antisocial behaviour amongover-reportingpatients**	**False negative predictions**
Study 1	0% (ASPD) 0% (SDAS-11 >1.5 *SD*s) 0% (LSRPS >57)	100% 100% 100%	ASPD: 0% SDAS-11 >1.5 *SD*: 0% LSRPS >57: 0%	100% 100% 100%
Study 2	0% (Intensive offender) 24% (LSRPS >57)	100% 76%	Intensive offenders: 0% LSRPS >57: 100%	100% 0%
Delain et al. (2003)	83% (ASPD)^a^	17%	ASPD: 17%	83%
Gacono et al. (1995)^b^	64% (ASPD)^a^ 90% (Psychopathy)^a^	36% 10%	ASPD: 100% Psychopathy: 100%	0% 0%
Heinze and Vess (2005)	11% (Psychopathy)	89%	Psychopathy: 33%^a^	67%
Kucharski, Duncan et al. (2006)	33% (Psychopathy)	67%	Psychopathy: 52%^a^	48%
Kucharski, Falkenbach et al. (2006)	31% (ASPD)	69%	ASPD: 72%^a^	28%

Note: ^a^Calculated using available information; ^b^Employed two samples: malingerers and non-malingerers. ASPD = antisocial personality disorder; LSRPS = Levenson Self-Report Psychopathy Scale; SDAS-11 = Social Dysfunction and Aggression Scale-11. Studies gathered from Niesten et al. (2015), and via a thorough search via Google Scholar using the search terms ‘psychopathy’ and ‘antisocial personality disorder’ combined with ‘malingering’, ‘feigning’, ‘simulation’, and ‘dissimulation’. Aside from the present studies, only studies that found a statistically significant relationship between symptom over-reporting and antisocial behaviour are included.

The vast variation in prevalence rates severely limits the predictive value of antisocial behaviour for symptom over-reporting. The lower the prevalence of symptom over-reporting relative to the prevalence of antisocial behaviour, the higher the false positive rate; and conversely, the lower the prevalence of antisocial behaviour relative to the prevalence of symptom over-reporting, the higher the false negative rate. As can be seen from Table 6, the employment of antisocial behaviour as a predictor of symptom over-reporting rarely yields acceptably low proportions of false positive and false negative predictions.

The weight of the evidence against the predictive utility of generic antisocial behaviour for symptom over-reporting is such that future research in this regard is uncalled for. Symptom over-reporting (including malingering) is better conceptualised as a context-dependent variable than as a trait that is associated with an antisocial disposition. Subsequent research should address specific forms of antisocial behaviour (e.g. subtypes of psychopathy or ASPD), particular contextual factors (e.g. subcategories of criminal or civil cases), and explicit patient characteristics (e.g. certain types of crime). For example, the present studies assess symptom over-reporting in post-trial settings where there are no incentives to feign symptoms that may affect the outcome of a trial (such as crime-related amnesia, dissociative fugue, command hallucinations, etc.). It may be that relations between malingering and antisocial or psychopathic behaviour are more pronounced among defendants than among convicts. Also, it may well be that associations exist between biased symptom reporting and antisocial or psychopathic behaviour by way of a third variable. For example, it is possible that antisocial and psychopathic individuals engage in more activities (e.g. criminal acts) that put them in situations (e.g. criminal trials) in which biased symptom-reporting is an appealing course of action. Furthermore, research could be strengthened by looking into multiple response styles (i.e. symptom over-reporting as well as under-reporting). While symptom over-reporting has often been discussed in relation to ASPD and psychopathy, symptom under-reporting has –undeservedly – received less attention.

Exaggerating symptoms to realise a transfer from prison to a psychiatric hospital is no less self-serving and deceptive than it is to conceal symptoms to secure an early release from a psychiatric ward. Thus, even if it is true that the occurrence of response bias is heavily dependent on certain character traits (e.g. psychopathic and antisocial traits), the form of response bias (i.e. positive or negative) remains largely dependent on contextual factors. A priori, then, an association with ASPD and psychopathy is as plausible for a positive response bias (e.g. symptom under-reporting) as it is for a negative response bias (e.g. symptom over-reporting). Hence, any assessment of the relation between malingering and psychopathic and antisocial traits is incomplete if it does not also address positive response bias. This should be a focus of subsequent research.

Study 2 found symptom over-reporting to be related to self-reported antisocial features, yet neither study found symptom over-reporting to be related to actual antisocial behaviour. This incongruity may be a clue to the discrepancies within the empirical literature about the association between symptom over-reporting and antisocial features, where a sizable portion found no association and an equally large portion found a small yet predictively trivial association.

The upshot of the preceding discussion is that antisocial behaviour is not a clinically useful indicator of symptom over-reporting or malingering. The *DSM-5* entry in this regard is scientifically untenable and its use leads to unacceptable rates of false-positive identifications of malingering in ill-disposed patients, and biases clinicians to miss cases of malingering among socially-accomplished individuals. Clinicians are strongly advised to refrain from using antisocial behaviour as a risk factor or indicator of symptom exaggeration. Instead, clinicians are urged – whether or not a patient presents with antisocial demeanour or meets criteria for ASPD or psychopathy – to *always* check for biased symptom-reporting by routinely including screening measures of symptom and performance validity in clinical assessments (Bush, Heilbronner, & Ruff, 2014).

## Notes

1. For scores of a certain index (e.g. the LSRPS, the SDAS-11, number of punitive actions) to have a diagnostic accuracy above chance level with regard to a dichotomous condition (e.g. symptom exaggeration, see note 3 below), the overlap between the index score distributions of the two conditions (e.g. honest and feigning) must be <50%. For example, if SDAS-11 scores are used as predictor of symptom exaggeration and the overlap between SDAS-11 scores of honest patients and feigning patients is 40%, then 60% of the patients can be classified correctly (i.e. 60% obtained scores that are unique to their group). Percentages of overlap between score distributions correspond to the magnitude of difference between the means of the score distributions (i.e. to Cohen's *d*; see Table 1 in Zakzanis, 2001). The sample size required for the conventional .80 statistical power to detect an effect that produces a diagnostic accuracy of at least 60% (i.e., *d* ≥ 1.2) is 54 when the base rate of symptom exaggeration is set at 10% (α = .05, one-tailed). The smallest groups in Studies 1 and 2 are the ones based on high SIMS scores (i.e. SIMS score >16; *n* = 5 and *n* = 6). The achieved power in comparisons of these groups with the groups that responded credibly to the SIMS is .81 for Study 1 and .85 for Study 2 (α = .05, one-tailed).

2. Studies were gathered from Niesten et al. (2015), and via a thorough search via Google Scholar with the search terms ‘psychopathy’ and ‘antisocial personality disorder’ combined with ‘malingering’, ‘feigning’, ‘simulation’, and ‘dissimulation’.
